# Quantifying the effect of isoflurane and nitrous oxide on somatosensory-evoked potentials

**DOI:** 10.4103/0019-5049.60496

**Published:** 2010

**Authors:** Usha Devadoss, S Babu, VT Cherian

**Affiliations:** Department of Anaesthesia, Christian Medical College, Vellore - 632 004, Tamilnadu, India; 1 Department of Neurosciences, Christian Medical College, Vellore - 632 004, Tamilnadu, India

**Keywords:** Brain tumour, monitoring, isoflurane, Propofol, SSEP

## Abstract

Anaesthetic techniques may have a significant effect on intraoperative-evoked potentials (EP). The present study is designed to compare Propofol anaesthesia with Isoflurane (with or without nitrous oxide) during intraoperative somatosensory-evoked potential (SSEP) monitoring in 15 ASA Grade I and II patients undergoing surgery for intracranial tumours. SSEPs in response to median and posterior tibial nerve stimulation were recorded under four different anaesthetic conditions: 1) Propofol infusion and ventilation with air-oxygen, 2) Isoflurane, 1.0 MAC and ventilation with air-oxygen, 3) Isoflurane 1.0 MAC and ventilation with nitrous oxide-oxygen, and 4) Return to Isoflurane, 1.0 MAC and ventilation with air-oxygen. Intraoperative monitoring of somatosensory evoked potentials is best recordable using Propofol. The morphology of the EP is reproducible with Isoflurane. This effect is exaggerated when it is advisable to avoid nitrous oxide.

## INTRODUCTION

**Figure 1:**
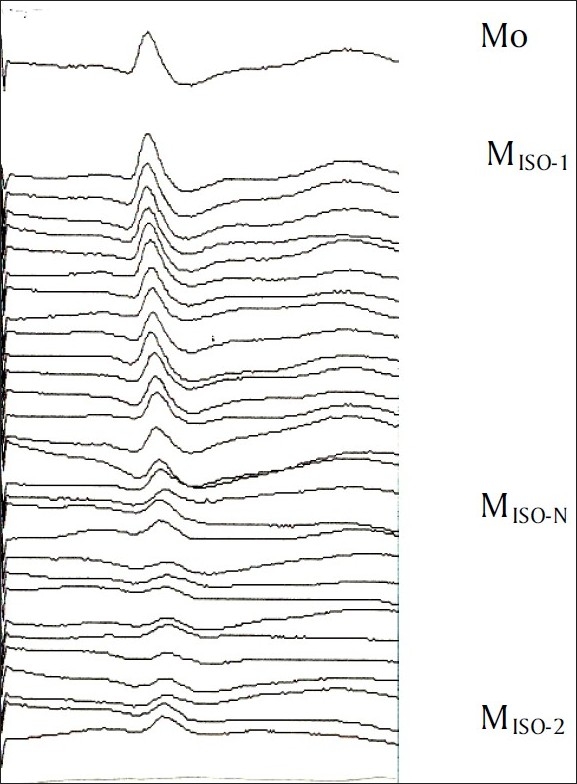
Serial recording of median nerve SSEP during study intervention in one patient

Monitoring of Somato Sensory Evoked Potential (SSEP) during surgery is to assess the functional integrity of the sensory pathways in an anaesthetised patient.[1,2] The latency and amplitude of the early cortical waves is usually monitored during surgery, namely the N20 for the median nerve and P37 for the posterior tibial nerve [Figure 1]. Although, intraoperative changes in SSEP are attributed to surgical trespass, physiological and pharmacological factors can cause temporary changes in their morphology.[1] Therefore, it is imperative to quantify the changes caused by the anaesthetic agents on SSEP to make it a useful tool to detect nerve damage during surgery. Total intravenous anaesthesia using Propofol is an acceptable technique for neurosurgery, but Isoflurane with or without nitrous oxide is still commonly used.[3] This study was undertaken to quantify the changes in the amplitude and latency of the early cortical SSEP caused by Isoflurane and nitrous oxide when compared to Propofol.

## METHODS

This study was approved by the research and ethics committee of our institution. All ASA Grade I or II patients scheduled for craniotomy and excision of a cerebral tumour were eligible to volunteer for this study. Patients with diabetes mellitus or lesions involving the primary sensory-motor cortex, those with significant preoperative abnormality of the evoked potentials, have been excluded from the study. The details of the study were explained and a written consent was taken from all who volunteered. The patients were premedicated with 10 mg of oral diazepam two hours prior to induction of anaesthesia. In the operating room, a long central venous catheter was inserted through the antecubital vein and a 20 G cannula into the radial artery, on the side, contra lateral to the one used for stimulation of the median nerve. The electrocardiogram, direct arterial pressure, temperature, oxygen saturation by pulse oximeter, end tidal (ET) concentrations of carbon dioxide, nitrous oxide and Isoflurane were monitored continuously. Anaesthesia was induced with an intravenous injection of midazolam (0.05 mg/kg), fentanyl (1-2 *μ*g/kg), Propofol (2-3 mg/kg) and vecuronium (0.1 mg/kg). The patients were ventilated with oxygen and air (FiO_2_-0.5) and the trachea was intubated using an appropriate sized endotracheal tube. Propofol was started at a rate of 10 mg/kg/hr for first 10 minutes, 8 mg/kg/hr for next 10 minutes and then continued at 6 mg/kg/hr. The site for the pins of the Mayfield head-clamp was infiltrated with 2% lignocaine.

The stimulating electrodes for the median and posterior tibial nerves were fixed at the wrist and the ankle respectively, on the same side as the lesion so that the evoked potential (EP) could be recorded on the side, contralateral to the side of surgery. The recording electrodes were placed on the scalp at C3' or C4' (median nerve) and at Cz (posterior tibial nerve), as per the ‘International 10-20’ system of electrode placement. The median and posterior tibial nerves were stimulated by a constant square pulse current of 20-35 mA for 200 *μ*sec at the rate of 4.7 Hz. Each recording was averaged over 300 stimuli.

### Study intervention

The median and posterior tibial nerve SSEPs were recorded alternately throughout the study period. The baseline values of the latency and the amplitude of the SSEP were noted once the Propofol infusion rate of 6 mg/kg/hr was reached and reproducible EP was obtained. This was done for both median (Mo) and posterior tibial nerve (To). Isoflurane was then added to the inspired gas mixture and the end tidal concentration of Isoflurane was monitored. The infusion of Propofol was tapered and stopped when the end tidal Isoflurane concentration reached 1%. The EP recorded at an end tidal Isoflurane of 1% was labelled as M_ISO-1_ or T_ISO-1_.

The oxygen-air mixture was then replaced by oxygen-nitrous oxide (FiO_2_-0.5) and serial EPs were recorded over the next 15 minutes. The EP at an end tidal nitrous oxide of 50%, along with an end tidal Isoflurane of 1%, was labelled as M_ISO-N_ or T_ISO-N_.

Finally, the nitrous oxide was turned off and air-oxygen mixture was re-instituted. Evoked potentials were recorded for another 10-15 minutes till the end tidal nitrous oxide became negligible. The EP waveform recorded at this stage was labelled as M_ISO-2_ or T_ISO-2_. All the data collection was completed before starting of the surgery.

### Data analysis

The amplitude and the latency of the early cortical EP waves, following stimulation of the median (N20) and the posterior tibial (P37) nerves, were noted at the following stages.

**Table:** 

	Median N	Posterior Tibial N
1. Baseline - Propofol air–O_2_	Mo	To
2. Air–O_2_ (FiO_2_ 0.5) + ET Isoflurane 1%	M_ISO-1_	T_ISO-1_
3. N_2_O–O_2_ (FiO_2_ 0.5) + ET Isoflurane 1%	M_ISO-N_	T_ISO-N_
4. Air–O_2_ (FiO_2_ 0.5) + ET Isoflurane 1%	M_ISO-2_	T_ISO-2_

The latency and the amplitude of N20 and P37 at Stage 2 and 3 were compared with those of the baseline. This difference was expressed as an absolute number and percentage change from the baseline so as to quantify the change in the EP waveform due to the addition of Isoflurane and nitrous oxide when compared to Propofol. Stages 2 and 3 were compared to quantify the additional effect of nitrous oxide and Stages 2 and 4 were compared to assess the residual effects of nitrous oxide. One-way repeated measure analysis of variance/Friedman's test was performed depending upon the normality of the data. Adjustment for multiple pair-wise comparisons was done using Bonferroni method. SPSS version 11.0 was used to carry out the analyses.

## RESULTS

Fifteen patients (eight women: seven men) who underwent craniotomy for excision of intracranial lesions, who consented were included in the study. Their age, weight, and height were 46.8±11.4 years, 56.1±8.8 kg and 158.7±9.6 cm respectively. The tumour was located in the cerebello-pontine angle in 13 patients and in the fronto-parietal region in two. The amplitude and latency of N20 following median nerve stimulation at M_O_ (Propofol), M_ISO-1_ (O_2_-air-Isoflurane), M_ISO-N_ (O_2_-N_2_ O-Isoflurane) and M_ISO-2_ (O_2_-air-Isoflurane) are shown in Table 1. The amplitude of N20 was significantly reduced when Isoflurane was substituted for Propofol (*P*-0.002) and was further reduced when nitrous oxide was administered (*P*-001). The latency of N20 was significantly prolonged with addition of Isoflurane (*P*-0.0001) and nitrous oxide (*P*-0.01) when compared to Propofol, but addition of nitrous oxide did not show any further prolongation in latency when compared with Isoflurane.

**Table 1:** The amplitude and latency of N20 following median nerve stimulation at M_O_ (Propofol), M_ISO-1_ (O_2_-air-Isoflurane), M_ISO-N_ (O_2_-N_2_O-Isoflurane) and M_ISO-2_ (O_2_-air-Isoflurane)

	M_O_	M_ISO-1_	M_ISO-N_	M_ISO-2_
Amplitude (μV)	5.50±4.11	3.23±2.40*P:0.002	1.95±1.56*P:0.001	2.63±2.02**
Latency (msec)	20.8±1.29	22.0±1.65*P: 0.000	22.07±1.61*P:0.010	22.52±1.84**

* Indicates - significant compared to baseline (M_O_)

** Indicates significant compared to M_ISO-1_

Similarly, the amplitude and the latency of P37 of the posterior tibial SSEP waveform at T_O_ (Propofol), T_ISO-1_ (O_2_-air-Isoflurane), T_ISO-N_ (O_2_-N_2_ O-Isoflurane) and T_ISO-2_ (O_2_-air-Isoflurane) are shown in Table 2. The baseline amplitude of P37 was 1.42±1.19 *μ*V and it was not significantly changed by Isoflurane but decreased with the addition of nitrous oxide (*P*-0.003). The latency of P37 was significantly prolonged by both Isoflurane and nitrous oxide (*P*-0.0001).

**Table 2:** The amplitude and latency of P37 following posterior tibial nerve stimulation at T_O_ (Propofol), T_ISO-1_ (O_2_-air-Isoflurane), T_ISO-N_ (O_2_- N_2_O- Isoflurane) and T_ISO-2_ (O_2_-air-Isoflurane) are shown

	T_O_	T_ISO-1_	T_ISO-N_	T_ISO-2_
Amplitude (μV)	1.42±1.19	1.16±1.32	0.61±0.63*	0.86±0.80
Latency (msec)	36.7±1.59	38.8±1.81*	39.9±1.91*	39.6±1.90

* (- Indicates statistical significance compared to baseline (T_O_))

The difference in the amplitude and latency of N20 and P37 at each stage of study intervention was calculated and expressed in absolute numbers and percentage change [Tables 3 and 4]. The introduction of Isoflurane decreased the amplitude of N20 by 41% and that of P37 by 32% and prolonged the latency of both by 5%. The combination of Isoflurane and nitrous oxide further decreased the amplitude of N20 to 63% and that of P37 to 56%, while the latency was prolonged to 7% and 8% respectively. The addition of nitrous oxide significantly decreased the amplitude of N20 and P37 as compared to when only Isoflurane was used and prolonged the latency of N20 by 2% and that of P37 by 4%.

**Table 3:** The difference in the amplitude and latency of N20 due to the effect of isoflurane and nitrous oxide

	Propofol-Isoflurane M_O_ - M_ISO-1_		Propofol-Iso + Nitrous oxide M_O_ - M_ISO1-N_		Isoflurane-Nitrousoxide M_ISO-1_ - M_ISO1-N_	
			
	Difference	% Change (95%CI)	Difference	% Change (95% CI)	Difference	% Change (95% CI)
Amplitude (μV)	−2.26±1.86	41.4±13.8 (33.7-49.1)	−3.55±2.70	63.2±13.4 (55.7-70.6)	−1.28±0.93	40.1±13.4 (32.6-47.5)
Latency (msec)	1.13±0.48	5.22±2.08 (4.0-6.3)	1.18±1.17	7.4±2.92 (5.78-9.02)	0.07±1.41	2.47±0.98 (1.97-3.02)

The difference is expressed in absolute numbers (negative sign denotes a decrease) and as percentage change

**Table 4:** The difference in the amplitude and latency of P37 due to the effect of isoflurane and nitrous oxide

	Propofol- isoflurane T_O_ - T_ISO-1_		Propofol- isoflurane+ nitrous oxide T_O_ - T_ISO1-N_		Isoflurane- nitrous oxide TISO-1 - T_ISO1-N_	
			
	Difference	% Change (95%CI)	Difference	% Change (95% CI)	Difference	% Change (95% CI)
Amplitude (μV)	−0.25±0.37	32.3±23.5 (18.7−45.8)	−0.81±0.66	56.4±20.9 (44.3−68.5)	−0.55±0.72	44.6±16.8 (34.8−54.3)
Latency (msec)	2.00±1.29	5.46±3.64 (3.3−7.5)	3.15±0.88	8.52±2.36 (7.15−9.88)	1.14±1.36	4.05±2.07 (1.97−3.02)

The difference is expressed in absolute numbers (negative sign denotes a decrease) and as percentage change.

The EPs were recorded after discontinuing nitrous oxide for about 10-15 minutes, when the end tidal nitrous oxide was negligible (M_ISO-2_, T_ISO-2_). Although, there was no significant difference in the amplitude and latency of P37 before and after nitrous oxide (T_ISO-1_ and T_ISO-2_) the decrease in amplitude and the prolongation in latency of N20 still persisted (*P*<0.05) as shown in Tables 1 and 2.

The mean blood pressure, heart rate, nasopharyngeal temperature and the end-tidal carbon dioxide, during Propofol infusion (baseline), O_2_-air-Isoflurane (ISO1), O_2_-N_2_ 0-Isoflurane (ISO-N), and O_2_-air-Isoflurane (ISO2) are shown in Table 5. Although, the heart rate and temperature showed significant (statistically) decrease when Isoflurane was used, it was not clinically significant.

**Table 5:** The mean blood pressure heart rate, nasopharyngeal temperature and end tidal carbon dioxide during the stages of study intervention

	Baseline	ISO1	ISO-N	ISO2
MBP (mm Hg)	90.0±15.3	84.20±18.2	75.87±13.2	80.67±14.5
Heart rate (per min.)	85.93±11.4	78.13±9.1	82.53±12.4	77.13±12.8*
Temperature °C	35.81±0.2	35.72±0.3	35.57±0.4*	35.45±0.4*
ETCO_2_ (mm Hg)	25.20±3.1	26.13±1.9	26.33±3.6	27.00±3.0

* (denotes statistically significant (*P*<0.05) difference from baseline, MBP:mean blood pressure

## DISCUSSION

Somatosensory Evoked Potential (SSEP) has become an integral part of monitoring during neurosurgery. It is the only method of continuously assessing the functional integrity of the sensory pathway in an anaesthetised patient. Unfortunately, apart from ischaemia and direct surgical damage to the neural tissues, frequently encountered changes in physiological factors and commonly used pharmacological agents can also affect the SSEP waveform. Since most of the anaesthetic agents in use today affect the SSEP, it becomes important to use an anaesthetic technique, which causes fewer changes and permits recording of acceptable waveforms. It is also essential to quantify the changes caused by such a technique to attribute any change apart from that to surgical trespass.[4]

This study is an attempt to quantify the changes caused by Isoflurane and nitrous oxide on SSEP, as compared to Propofol. The recommended infusion rate for Propofol was used to obtain the baseline waveform[5] Since the cortical SSEP waveform take about 5-8 minutes to stabilise after any change in volatile anaesthetic concentration,[6] the measurements were noted from waveforms obtained five minutes after the steady state of the required ET concentration.

Isoflurane, in a concentration of 1% (0.8 MAC[7,8]), reduced the amplitude of N20 by about 40% compared to the baseline, and prolonged the latency by 5%, which is comparable to the findings of other studies.[9,10] The absolute decrease in amplitude was found to be about 2.26 *μ*V and the increase in latency was about 1 m sec, which would be a more useful information while interpreting intra-operative changes in EP. The amplitude of P37 was reduced by 0.25 μV (30%) and the latency increased by 2 m sec (5%).

The decrease in the amplitude and the increase in the latency of early cortical waveform were exacerbated by the addition of nitrous oxide. The administration of nitrous oxide with Isoflurane decreased the amplitude of N20 to 3.5 *μ*V (63%) and increased their latency by 1.1 m sec (7%) as compared to when only Propofol was used. Addition of nitrous oxide decreased the amplitude of P37 to 0.8 μV (56%) and increased their latency by 3 m sec (8%). This change could be partly due to the increased anaesthetic potency of the combination of 1% Isoflurane with 50% nitrous oxide (1.2 MAC) as compared to only 1% Isoflurane.

It has been shown that increasing concentrations of Isoflurane causes a graded reduction in amplitude and increase in latency of EP.[9] Nitrous oxide, by itself, has been shown to have no effect on the latency of cortical evoked potentials,[11,12] but it has been shown to have additive effect at higher concentrations.[9] This study has shown that nitrous oxide reduces the amplitude by additional 40%. Therefore, it is advisable to avoid nitrous oxide in patients where monitoring of EP is crucial, especially with low voltage baseline waveforms.

Due to the study design, the effect of Propofol on the amplitude and the latency of SSEP waveform could not be ascertained. However, the amplitude of the EP was highest and latency shortest, when maintained on Propofol infusion, as demonstrated by other studies.[13–15]

Plasma concentrations of Propofol declined more rapidly after the end of infusion, the redistribution half-lives were for 13.4 minutes.[16]

Since physiological parameters such as temperature, end tidal carbon dioxide, and mean arterial pressure can influence SSEP waveforms,[1,18] it was monitored continuously during the study period. It was noted during the later part of the study intervention, the heart rate was slower and the temperature was lower. This was of no clinical significance. Therefore, the changes in amplitude and latency, seen during the study intervention, are not likely to have been influenced by the physiological parameters. Since most clinical neurophysiologists would consider a decrease in amplitude of 50% or more, or an increase in latency of 10% or more to be significant, reflecting loss of integrity of a neural pathway,[1] it would seem prudent to avoid an anaesthetic technique which causes these levels of changes.

During intra-operative SSEP monitoring to detect neuronal damage, more importance is usually attributed to a decrease in amplitude rather than a prolongation in latency of EP. However, two observations were made in this study. First, when the baseline waveforms of all the study patients were compared, the variability in the amplitude was higher (5.5±4.11 *μ*V) than that of the latency (20.81±1.29 m sec). Second, the effect of Isoflurane and nitrous oxide was more on the amplitude than on the latency. Hence, one would wonder if latency is a more stable criterion to observe than the amplitude.

According to this study, nitrous oxide should be avoided when intra-operative monitoring of SSEP is intended. Although, the changes caused by 1% Isoflurane were significant, reproducible, acceptable waveforms were recorded in all the patients and therefore it is an acceptable anaesthetic technique. However, the best SSEP waveforms were recorded during total intravenous anaesthesia with Propofol; it is recommended for surgery where EP monitoring is crucial. The lack of pollution of the operating room; unlike with volatile anaesthetic, reduced incidence of PONV, rapid redistribution possibly reflected in the superior clinical recovery, are additional advantages with a total intravenous anaesthetic technique with Propofol.

## References

[1] Banoub M, Tetzlaff JE, Schubert A (2003). Pharmacologic and physiologic influences affecting sensory evoked potentials. Anesthesiology.

[2] Chiappa KH, Ropper AH (1982). Evoked potentials in clinical medicine. N Engl J Med.

[3] Taniguchi M, Nadstawek J, Pechstein U, Schramm J (1992). Total intravenous anaesthesia for improvement of intra-operative monitoring of somato-sensory evoked potentials during aneurysm surgery. Neurosurgery.

[4] Peterson DO, Drummond JC, Todd MM (1986). Effects of halothane, enflurane, isoflurane and nitrous oxide on somato-sensory evoked potentials in humans. Anesthesiology.

[5] Mirakhur RK, Morgan M (1998). Intravenous anaesthesia: a step forward. Anaesthesia.

[6] Mason DG, Higgins D, Boyd SG, Lloyd-Thomas AR (1992). Sequential measurement of the median nerve somatosensory evoked potential during Isoflurane anaesthesia in children. Br J Anaesth.

[7] Mapleson WW (1998). The theoretical ideal fresh-gas flow sequence at the start of low-flow anaesthesia. Anaesthesia.

[8] Aitkenhead AR, Rowbotham DJ, Smith G (2001). Text book of anaesthesia.

[9] Sebel PS, Ingram DA, Flynn PJ, Rutherfoord CF, Rogers H (1986). Evoked potentials during Isoflurane anaesthesia. Br J Anaesth.

[10] Lam AM, Sharar SR, Mayberg TS, Eng CC (1994). Isoflurane compared with nitrous oxide anaesthesia for intraoperative monitoring of somatosensory evoked potentials. Can J Anaesth.

[11] Sloan TB, Koht A (1985). Depression of cortical somatosensory evoked potentials by nitrous oxide. Br J Anaesth.

[12] Sebel PS, Flynn PJ, Ingram DA (1984). Effect of nitrous oxide on visual, auditory and somatosensory evoked potentials. Br J Anaesth.

[13] McPherson RW, Mahla M, Johnson R, Traystman RJ (1985). Effects of enflurane, Isoflurane and nitrous oxide on somatosensory evoked potentials during fentanyl anaesthesia. Anesthesiology.

[14] Grundy BL (1983). Intraoperative monitoring of sensory-evoked potentials. Anesthesiology.

[15] Kalkman CJ, Traast H, Zuurmond WW, Bovill JG (1991). Differential effects of prorofol and nitrous oxide on posterior tibial nerve somatosensory cortical evoked potentials during alfentanil. anaesthesia. Br J Anaest.

[16] McMurray TJ, Collier PS, Carson IW, Lyons SM, Elliott P (1990). Propofol sedation after open heart surgery. A clinical and pharmacokinetic study. Anaesthesia.

[17] Cockshott ID, Briggs LP, Douglas EJ, White M (1987). Pharmacokinetics of propofol in female patients. Br J Anaesth.

[18] Kumar A, Bhattacharya A, Makhija N (2000). Evoked potential monitoring in anaesthesia and analgesia. Anaesthesia.

